# Supplementary material for: The Systems Biology Research Tool: evolvable open-source software
Source: BMC Syst Biol. 2008 Jun 29;2:55. doi: 10.1186/1752-0509-2-55 (PMC2446383; doi:10.1186/1752-0509-2-55)
Supplement: Additional file 1 — SBRT Archive. An archive of the current version of the Systems Biology Research Tool. [file 1752-0509-2-55-S1.zip › sbrt-1.4.0/doc/developers_guide/api/sbrt/shell/text/package-use.html]

Uses of Package sbrt.shell.text


|  |  |  |
| --- | --- | --- |
| **Overview** \| **Package** \| Class \| **Use** \| **Tree** \| **Deprecated** \| **Index** \| **Help** | |  |
| PREV   NEXT | **FRAMES**    **NO FRAMES**     **All Classes** |  |


---


## **Uses of Package sbrt.shell.text**

| Packages that use sbrt.shell.text | |
| --- | --- |
| **sbrt.shell.io** | Provides classes and interfaces for reading and writing files. |
| **sbrt.shell.io.fba** | Provides classes and interfaces for reading and writing files relevant to Flux Balance Analysis. |
| **sbrt.shell.mng** | Provides classes and interfaces for creating process managers. |
| **sbrt.shell.mng.fba** | Provides classes and interfaces for creating Flux Balance Analysis process managers. |
| **sbrt.shell.mng.geom** | Provides classes and for creating geometry process managers. |
| **sbrt.shell.proc.fba** | Provides classes for converting FBA-related files of different formats. |
| **sbrt.shell.text** | Provides classes and interfaces for defining formats for basic objects. |
| **sbrt.shell.text.chem** | Provides classes and interfaces for defining formats for chemistry related objects. |
| **sbrt.shell.text.fba** | Provides classes and interfaces for defining formats for objects relevant to Flux Balance Analysis. |
| **sbrt.shell.text.math** | Provides classes and interfaces for defining formats for mathematical objects. |

Classes in sbrt.shell.text used by sbrt.shell.io

****AppInputFileLineFormat****
  
          This interface is used to represent the format of
lines in process input files.

****Formatter****
  
          This interface is used to represent *formatters*.

****MapFormat****
  
          This interface is used to represent formats for maps.

****Parser****
  
          This interface is used to represent *parsers*.

Classes in sbrt.shell.text used by sbrt.shell.io.fba

****MapFormat****
  
          This interface is used to represent formats for maps.

Classes in sbrt.shell.text used by sbrt.shell.mng

****PercentFormat****
  
          This interface is used to represent a format for
percentages.

Classes in sbrt.shell.text used by sbrt.shell.mng.fba

****MapFormat****
  
          This interface is used to represent formats for maps.

Classes in sbrt.shell.text used by sbrt.shell.mng.geom

****MapFormat****
  
          This interface is used to represent formats for maps.

Classes in sbrt.shell.text used by sbrt.shell.proc.fba

****EqualsMapEntryFormatV1****
  
          This class is a concrete implementation of `MapEntryFormat`.

Classes in sbrt.shell.text used by sbrt.shell.text

****AbstractLinkedHashSetFormat****
  
          This abstract class is a skeleton implementation of
`LinkedHashSetFormat`.

****AbstractListFormat****
  
          This abstract class is a skeleton implementation of `ListFormat`.

****AbstractMapEntryFormat****
  
          This abstract class is a skeleton implementation of
`MapEntryFormat`.

****AbstractMapFormat****
  
          This abstract class is a skeleton implementation of
`MapFormat`.

****AmpersandSetFormat****
  
          This class is used to define a format for ampersand-delimited sets.

****AppInputFileLineFormat****
  
          This interface is used to represent the format of
lines in process input files.

****BooleanFormat****
  
          This interface is used to represent a format for boolean
values.

****BoolRelFormatV1****
  
          This interface is used to represent boolean relationship
formats.

****ColonMapEntryFormatV1****
  
          This class is a concrete implementation of `MapEntryFormat`.

****ColonMapFormatV1****
  
          This class is a concrete implementation of `MapFormat`.

****DoubleFormat****
  
          This interface is used to represent a format for double
precision numbers.

****DoubleFormatV1****
  
          This class is a concrete implementation of `DoubleFormat`.

****EqualsMapEntryFormatV1****
  
          This class is a concrete implementation of `MapEntryFormat`.

****EqualsMapFormatV1****
  
          This class is a concrete implementation of `MapFormat`.

****Format****
  
          This interface is used to represent formats.

****Formatter****
  
          This interface is used to represent *formatters*.

****IntegerFormat****
  
          This interface is used to represent a format for integers.

****LinkedHashSetFormat****
  
          This interface is used to represent `LinkedHashSet`
formats.

****ListFormat****
  
          This interface is used to represent list formats.

****LongFormat****
  
          This interface is used to represent a format for longs.

****MapEntryFormat****
  
          This interface is used to represent formats for map entries.

****MapFormat****
  
          This interface is used to represent formats for maps.

****Parser****
  
          This interface is used to represent *parsers*.

****PercentFormat****
  
          This interface is used to represent a format for
percentages.

****PipeListFormat****
  
          This class is used to define a format for pipe-delimited
lists.

****PipeSetFormat****
  
          This class is used to define a format for pipe-delimited
sets.

****SimpleFormat****
  
          This interface is used to represent simple formats.

****TabListFormat****
  
          This class is used to define a format for tab-delimited
lists.

****TabSetFormat****
  
          This class is used to define a format for tab-delimited
sets.

****WhitespaceSetFormat****
  
          This class is used to define a format for whitespace-delimited
sets.

Classes in sbrt.shell.text used by sbrt.shell.text.chem

****Format****
  
          This interface is used to represent formats.

****Formatter****
  
          This interface is used to represent *formatters*.

****Parser****
  
          This interface is used to represent *parsers*.

****SimpleFormat****
  
          This interface is used to represent simple formats.

Classes in sbrt.shell.text used by sbrt.shell.text.fba

****AbstractMapFormat****
  
          This abstract class is a skeleton implementation of
`MapFormat`.

****ColonMapEntryFormatV1****
  
          This class is a concrete implementation of `MapEntryFormat`.

****DoubleFormatV1****
  
          This class is a concrete implementation of `DoubleFormat`.

****EqualsMapEntryFormatV1****
  
          This class is a concrete implementation of `MapEntryFormat`.

****Format****
  
          This interface is used to represent formats.

****Formatter****
  
          This interface is used to represent *formatters*.

****MapFormat****
  
          This interface is used to represent formats for maps.

****Parser****
  
          This interface is used to represent *parsers*.

****PipeListFormat****
  
          This class is used to define a format for pipe-delimited
lists.

****PipeSetFormat****
  
          This class is used to define a format for pipe-delimited
sets.

****SimpleFormat****
  
          This interface is used to represent simple formats.

****WhitespaceListFormat****
  
          This class is used to define a format for whitespace-delimited
lists.

Classes in sbrt.shell.text used by sbrt.shell.text.math

****DoubleFormatV1****
  
          This class is a concrete implementation of `DoubleFormat`.

****Format****
  
          This interface is used to represent formats.

****Formatter****
  
          This interface is used to represent *formatters*.

****Parser****
  
          This interface is used to represent *parsers*.

****SimpleFormat****
  
          This interface is used to represent simple formats.

---


|  |  |  |
| --- | --- | --- |
| **Overview** \| **Package** \| Class \| **Use** \| **Tree** \| **Deprecated** \| **Index** \| **Help** | |  |
| PREV   NEXT | **FRAMES**    **NO FRAMES**     **All Classes** |  |


---
